# Between a Rock and a Hard Place: Percutaneous Aspiration and Debulking for Tricuspid Valve Endocarditis

**DOI:** 10.7759/cureus.25166

**Published:** 2022-05-20

**Authors:** Gerry S Eichelberger, Maria Kocab, Reinaldo Claudio, Kellee L Oller

**Affiliations:** 1 Internal Medicine, University of South Florida Morsani College of Medicine, Tampa, USA; 2 Medicine, University of South Florida Morsani College of Medicine, Tampa, USA

**Keywords:** angiovac and infective endocarditis, intravenous drug use (ivdu), angiovac and debulking of tricuspid valve endocarditis, angiovac, right sided infective endocarditis, tricuspid, infective endocarditis

## Abstract

Right-sided infective endocarditis (IE) constitutes about 10% of total IE cases. Of these, tricuspid endocarditis comprises about 90% of all right-sided IE cases with intravenous drug use (IVDU) as its strongest risk factor. In patients with larger vegetations (>20 mm) or with persistent bacteremia, surgical intervention is often the standard of care. With FDA approval in 2014 and limited cases with regards to its application in tricuspid endocarditis, AngioVac (AngioDynamics, Latham, NY) has been used as a less invasive, off-label, bridging agent for tricuspid IE treatment. We present a case of a 40-year-old man with a past medical history of IVDU who presented with tricuspid endocarditis. His blood cultures were positive for methicillin-susceptible *Staphylococcus aureus* bacteremia. A transthoracic echocardiogram showed a 2.7 x 1.1 cm vegetation of the tricuspid valve. The patient was thought to be a poor surgical candidate for multifactorial reasons including patient preference, hemodynamic instability, and a hospital course that was complicated by septic emboli and infectious glomerulonephritis. The patient was unable to clear blood cultures despite appropriate antibiotic therapy. He subsequently underwent an AngioVac procedure with removal of the vegetation from his tricuspid valve achieving adequate source control, clear blood cultures, and resolution of endocarditis.

As this case illustrates, AngioVac should be considered an effective alternative to surgical intervention in tricuspid endocarditis. Further research and awareness of the utility of AngioVac in right-sided endocarditis are warranted and should be conducted.

## Introduction

Extensive research regarding left-sided endocarditis exists in the literature; however, there is much less published regarding right-sided endocarditis. Current treatment of right-sided endocarditis includes medical management for small vegetations and more aggressive measures for large vegetations, most often surgical intervention [1,2]. AngioVac (AngioDynamics, Latham, NY) is a vacuum-based percutaneous approach that is at this moment approved to remove things such as thrombi, emboli, and unwanted materials from the body, though recently it has been used off-label for vegetations [3]. As it stands today, guidelines suggest that surgery is indicated in patients with vegetations > 1 cm on the mitral or aortic valve and embolic events or in the presence of persistent infection. However, the significance of vegetation size in right-sided infective endocarditis (IE) is less defined. Studies show that in right-sided infection endocarditis, a vegetation size of >20 mm was associated with higher mortality when compared with a vegetation size of ≤20 mm [2,4]. In right-sided IE in drug users, vegetations > 20 mm and fungal infection have been associated with high in-hospital mortality [5,6]. In summation, sufficient data are not available on right-sided endocarditis cases that carry an all-cause late mortality and those with vegetations > 20 mm, with even more limited management options.

## Case presentation

A 40-year-old man with a history of hepatitis C and intravenous drug use (IVDU) presented with worsening dyspnea, chest pain, myalgias, and fever for three weeks. On exam, he was ill-appearing with bibasilar crackles on auscultation and a 2/6 systolic murmur heard best at the left lower sternal border. Transthoracic echocardiogram (TTE) demonstrated a single (1.9 x 0.7 cm) mobile mass on the right atrial aspect of the tricuspid valve with severe tricuspid regurgitation (TR). Computed tomography angiography of the chest demonstrated bilateral segmental pulmonary emboli and pulmonary infarcts in the left lung. Empiric broad-spectrum antibiotics were started. His blood cultures yielded methicillin-susceptible *Staphylococcus aureus* (MSSA) that was sensitive to cefazolin and the patient was transitioned to IV cefazolin. He was found to be acutely and severely septic and transferred to the intensive care unit for further management. During admission, the patient developed worsening renal function secondary to infectious glomerulonephritis and he remained persistently bacteremic despite appropriate medical therapy with IV antibiotics. Cardiothoracic surgery was consulted; however, the patient desired avoidance of surgery and was deemed a poor surgical candidate due to hemodynamic instability (hypotensive and tachycardic). Interventional radiology was consulted for possible AngioVac procedure. A repeat TTE was performed and revealed vegetation larger than the prior study (2.7 x 1.1 cm; Video 1).

**Video 1:** Echocardiogram with tricuspid valve vegetation (2.7 x 1.1 cm)

**Figure 1:**
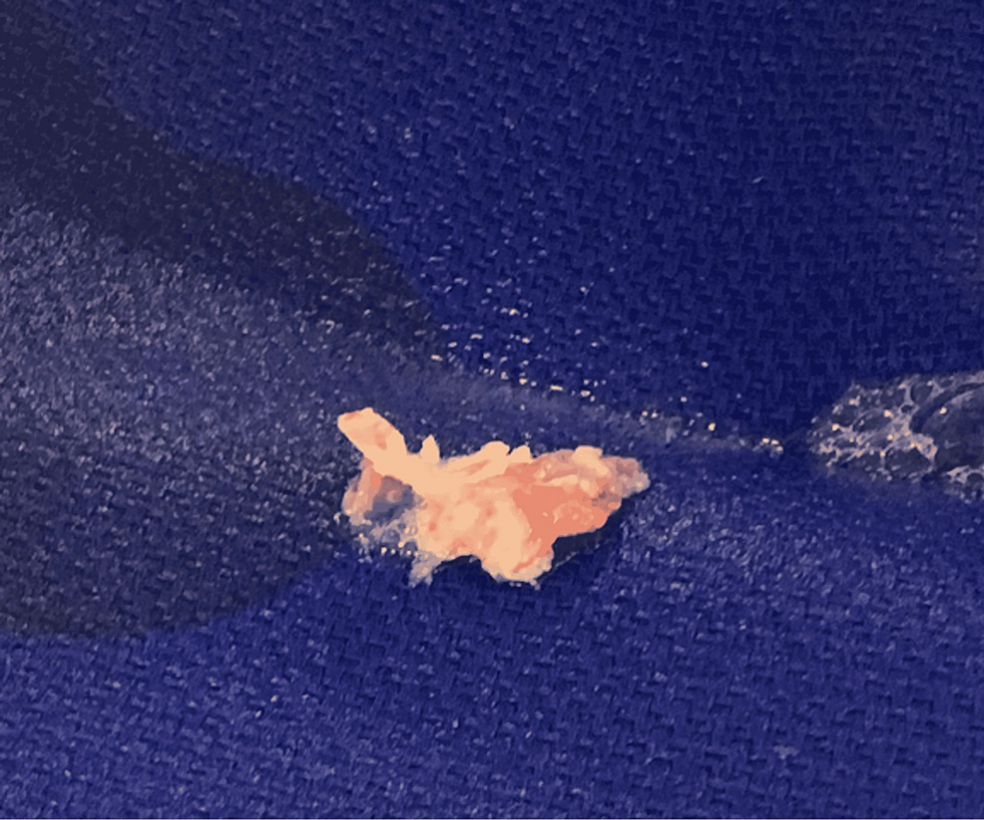
Image of aspirated vegetation (2.7 x 1.1 cm)

The patient was taken to the interventional radiology suite and was sedated. Under transesophageal echocardiography (TEE) guidance, the vegetation was suctioned and extracted. He successfully underwent this minimally invasive procedure, percutaneous aspiration, and debulking of the vegetation of the tricuspid valve, to provide source control without complications. A picture of the aspirated vegetation is shown below (Figure 1) along with echocardiogram findings immediately after the removal of the vegetation (Video 2).

**Video 2:** Echocardiogram following AngioVac, with vegetation removed

After this procedure was performed, the patient’s overall condition improved. Blood cultures were subsequently negative. The patient continued with IV cefazolin to complete a course of six weeks of antibiotic therapy and showed adequate recovery post-procedure.

## Discussion

While left-sided endocarditis indications for surgery are well established, the role is less clear in right-sided endocarditis. Surgery is often employed in situations of right-sided endocarditis with right-sided heart failure, moderate to severe tricuspid regurgitation with poor medical therapy response, multiresistant bacteria, or large vegetations greater than 20 mm [2].

We describe a case of tricuspid endocarditis successfully treated and managed with aid of the AngioVac procedure due to poor surgical candidacy and patient preference. AngioVac is currently not approved by the FDA for use in right-sided endocarditis. However, our patient successfully underwent debulking of his right-sided 2.7 x 1.1 cm vegetation, which ultimately led to subsequent negative blood cultures despite the previous failure to source control prior to the procedure. The patient had an uncomplicated postoperative course.

AngioVac serves as a non-invasive approach that can be used especially in critically ill patients who are at high operative risk. Traditionally, AngioVac has been used in iliocaval, pulmonary, and right-sided heart chamber thrombi. The concept behind using AngioVac in right-sided IE stems from the idea of ultimately diminishing vegetation size and thus bacterial load. This in turn allows antibiotics to attack a lesser bacterial load, thus effectively clearing the bloodstream infection. High bacterial inoculums are associated with higher antibiotic resistance and reduced penetration, a phenomenon known as the inoculum effect [7].

In a review of current medical literature, there are limited demonstrations of AngioVac application in tricuspid valve vegetations. George et al. (2017) performed the largest study in current research, i.e., a retrospective study on 33 patients (14 methicillin-resistant *Staphylococcus aureus* (MRSA), 11 MSSA, three polymicrobial, and five *Candida*) in which the subset of patients undergoing AngioVac had lacked a response to appropriate antimicrobial therapy or medical management alone [8]. The average vegetation size for the study was 2.1 ± 0.7 cm and AngioVac debulking resulted in an average 61% reduction in the size of the vegetation or a 1.3 cm reduction in size on echocardiogram. The endocarditis resolved in 28/33 patients with no further complications. All patients survived the procedure with 90.9% surviving hospitalization. The only post-procedural complication of note was in one patient who developed cardiac tamponade requiring pericardiocentesis. Of the three documented patient deaths that occurred several days to weeks after the procedure, one involved a young male with a previous bioprosthetic tricuspid valve infected with polymicrobial organisms inclusive of MRSA, *Enterococcus faecium*, *Acinetobacter baumannii*, and *Candida glabrata,* who injected drugs intravenously during the hospitalization. The second was a patient with extensive epidural abscess leading to spinal cord compression and spinal cord edema. The last patient's death was from other causes of aortic valve perforation and spinal osteomyelitis. Another retrospective study conducted with a sample size of 20 was done by Schaerf et al. (2016), in which AngioVac was used in this patient population for a similar lack of response to appropriate antimicrobial therapy, but also as an adjunct or bridge to percutaneous lead removal in some cases. The vegetations in this study were larger 3.6 ± 1.2 cm. Endocarditis in 19/20 of these resolved. Complications were not well reported in this study [9]. Overall, the research and data are severely lacking especially with regard to studies of large sample sizes in which patients have undergone AngioVac debulking for right-sided endocarditis.

Expanding the role of AngioVac as an alternative to surgery and defining criteria and indications for possible candidates is necessary for the future of right-sided endocarditis management. This begins with awareness of this application of AngioVac and increasing knowledge of successful cases of tricuspid endocarditis debulking such as in this case. This case depicts the importance of employing appropriate clinical judgment and considering alternative means to provide source control in bacteremic patients with right-sided endocarditis.

## Conclusions

The AngioVac procedure has largely been used in the aspiration of iliocaval, pulmonary, upper extremity, and right-sided heart chamber thrombi, but with very few studies on the efficacy for tricuspid IE treatment. There are no set criteria for who would benefit the most from this procedure for right-sided IE. It is presently used as an alternative therapy for those who meet the criteria for surgery but are not surgical candidates. When patients with tricuspid IE are either not amenable to surgery or are not surgical prospects, AngioVac serves as a less invasive and efficacious treatment option to surgery and should be studied further to determine candidacy criteria. With further research, advancement in this technique and procedure can perhaps allow for the definition of guidelines to determine the best use or pre-surgical uses.
